# Smooth Muscle α-Actin Expression in Mitral Valve Interstitial Cells is Important for Mediating Extracellular Matrix Remodeling

**DOI:** 10.3390/jcdd7030032

**Published:** 2020-08-19

**Authors:** Bailey K. Dye, Catalina Butler, Joy Lincoln

**Affiliations:** 1 Biomedical Sciences Graduate Program, The Ohio State University, Columbus, OH 43217, USA; dye.210@osu.edu; 2 Department of Pediatrics, Medical College of Wisconsin, Milwaukee, WI 53226, USA; 3 Division of Pediatric Cardiology, The Herma Heart Institute, Children’s Wisconsin, Milwaukee, WI 53226, USA; 4 Harvard College, Boston, MA 02138, USA; cbutler@college.harvard.edu

**Keywords:** heart valve, extracellular matrix, heart valve interstitial cells, myxomatous degeneration

## Abstract

Background: Mitral valve prolapse (MVP) affects 3–6% of the total population including those with connective tissue disorders. Treatment is limited, and patients commonly require surgery which can be impermanent and insuperable. Abnormal prolapse of mitral valve leaflets into the left atria is caused by disturbances to the composition and organization of the extracellular matrix (ECM), that weaken biomechanics. This process, known as myxomatous degeneration is characterized by an abnormal accumulation of proteoglycans, in addition to collagen fiber disruption and elastic fiber fragmentation. The underlying mechanisms that promote myxomatous degeneration to the point of biomechanical failure are unknown, but previous histological studies of end-stage diseased tissue have reported abnormal α-smooth muscle actin (SMA) in a subset of heart valve interstitial cells (VICs); however, the contribution of these abnormal cells to MVP pathogenesis has not been extensively examined. Methods: In vivo and in vitro approaches were used. Mice harboring a *Fbn1^C1039G^* mutation mimic human Marfan Syndrome and develop MVP. Using these mice, temporal and spatial changes in SMA expression relative to myxomatous degeneration were examined using histological techniques. In parallel in vitro experiments, SMA expression was downregulated in primary porcine mitral VICs directly using siRNA, and indirectly using the actin depolymerizing agent Latrunculin A. In addition, the regulation of SMA in VICs by mechanical stiffness was explored relative to ECM remodeling. Results: We show, in mitral valves from *Fbn1^C1039G/+^* mice, that abnormal increases in SMA expression in VICs are evident during early postnatal stages of disease, prior to significant myxomatous degeneration as indicated at later stages by increased proteoglycans and collagen type I (Col1a1). Furthermore, abnormal SMA expression continues to increase during the course of pathogenesis and is localized to the mid belly region of the mitral valve leaflets from 10 weeks. Using an in vitro approach, we demonstrate that reduced SMA function by direct siRNA or indirect Latrunculin A treatment attenuates proteoglycan and Col1a1 expression in porcine mitral VICs. While upstream, we provide insights to show that SMA is regulated by mechanical tension in VICs to promote changes in ECM homeostasis. Conclusions: Together, our data show that in VICs, SMA, an actin binding protein, is important for mediating ECM remodeling associated with phenotypes observed in myxomatous degeneration, and its expression is regulated by mechanical tension. These novel insights could inform the development of future non-surgical therapeutics to halt the progression of mitral valve degeneration thereby avoiding end-stage prolapse.

## 1. Introduction

The mitral valve leaflets open to allow blood to flow from the left atria into the left ventricle, and close tightly to prevent backflow. This coordinated opening and closing is largely facilitated by a dynamic and diversified connective tissue system that provides all the necessary biomechanical properties to withstand constant changes in the hemodynamic environment [1]. The extracellular components of the valve leaflets include three highly organized layers of specialized matrix. The fibrosa layer is situated furthest away from the flow on the ventricular surface and predominantly comprises parallel bundles of fibrillar collagens that provide tensile strength; the spongiosa is rich in proteoglycans with a lower abundance of collagens and thereby provides a more compressible matrix; while the atrialis adjacent to blood flow contains mostly elastic fibers and allows for flexibility and extensibility [2]. It is the composition and order of these matrix layers that provide the necessary biomechanics to allow the leaflets to fully extend open during diastole, and tightly close during systole. Formation of the highly organized extracellular matrix (ECM) structure begins in the embryo, as endocardial cushions physiologically remodel into valve primordia, and this process is believed to be mediated by valve interstitial cells (VICs) that express high levels of α-smooth muscle actin (SMA) during this stage of development. Stratification of the ECM layers continues during the first few weeks of postnatal maturation and subsides as VICs downregulate SMA. VICs remain in this quiescent state throughout life in the absence of disease and serve to mediate ECM turnover by regulating a fine balance between degradation and secretion in response to normal wear-and-tear [3,4]. The VIC population is critical in ensuring that the structure, and therefore biomechanical function of the mitral valve leaflets is maintained throughout life.

Mitral Valve Prolapse (MVP) is a common valvulopathy affecting 3–6% of the population, and the most common cause of primary mitral regurgitation requiring surgery [5,6]. MVP includes syndromic forms as seen in connective tissue disorders including Marfan Syndrome, and more commonly non-syndromic forms which may carry a genetic component, but valvular phenotypes are isolated (reviewed [7]). While mild MVP is not necessarily life threatening, if untreated, symptoms can worsen and lead to congestive heart failure and sudden death. MVP is largely characterized by increased area and length of mitral valve leaflets, which appear floppy, with one or both leaflets prolapsing back into the left atrium as a result of ECM disturbances related to myxomatous degeneration [8,9]. Histologically, this phenotype includes expansion of the spongiosa layer due to an abnormal accumulation of glycosaminoglycans (GAGs) and proteoglycans including decorin, biglycan, and versican [9,10], as well as collagen fiber disruption, elastic fiber fragmentation, and increased expression of matrix metalloproteinases (MMPs) [9,11,12,13,14,15,16,17]. As a result, the net change is an overall increase in ECM, which causes the leaflets to thicken, and over time biomechanically weaken leading to a global reduction in tissue stiffness [18,19]. By the end-stage, hooding or doming of the leaflets back into the left atria can be viewed by echocardiography, and if severe, patients are referred for surgical intervention within one year, and this was the treatment path for over 180,000 Americans in 2018.

The underlying mechanisms of myxomatous degeneration in MVP are unknown but insights have been gained from mouse models of syndromic human disease. Perhaps the most well studied is the *Fbn1^C1039G^* mouse that harbors a cysteine to glycine knock-in mutation in *Fibrillin-1* and recapitulates features of Marfan Syndrome [20]. In affected humans, dissection of predisposed aortic aneurysms poses the greatest life-threatening risk; however, MVP is highly prevalent, affecting 43% of patients by 30 years of age, and 77% by the age of 60 [21], highlighting the longitudinal severity. Mice homozygous for *Fbn1^C1039G/C1039G^* die between postnatal day (PND) 7–10 from aortic dissection [22], while heterozygotes (*Fbn1^C1039G/+^*) are viable, but show progressive deterioration of the aortic wall [20]. Mitral valves from *Fbn1^C1039G/+^* mice show significant thickening and progressively develop systolic prolapse by 9 months [20], suggesting that similar to humans, mitral valve disease advances over time. The nature of the *C1039G* mutation has been shown to largely not affect Fbn1 expression, but rather impairs microfibril formation [22]. Furthermore, the mutation leads to the inability of functional FBN1 to bind TGFβ, leading to a global increase in circulating TGFβ levels, and TGFβ activity in affected tissues [20,23]. Treatment of pregnant *Fbn1^C1039G/+^* dams with a TGFβ neutralizing antibody prevents mitral valve phenotypes after birth and lowers serum levels [20,23]. However, in humans, favorable outcomes following treatment with TGFβ inhibitors such as Losartan remain unsubstantiated, and improvement of the mitral valve function has not been extensively studied (reviewed [23,24]). While *FBN1* mutations and subsequent increases in TGFβ signaling cause the initial onset of mitral valve pathogenesis in Marfan Syndrome, the mechanisms that further drive progressive myxomatous degeneration to the point of end-stage prolapse are not known.

Histological studies of myxomatous mitral valves from affected humans and susceptible dog breeds have identified the abnormal presence of SMA-positive VICs within the affected tissue at the end-stage [9,25,26,27,28]. Based on this molecular profile, these VICs have been termed “activated” and designated for their myofibroblast-like characteristics [29]. The role of SMA-positive “activated” VICs in myxomatous degeneration is not known, but work by Rabkin et al., describes an association with matrix remodeling genes including MMPs, cathepsins, and TIMPs [9], suggestive of pathological remodeling. In this study, we address current deficits in the literature and further explore the contribution and regulation of abnormal SMA-positive cells in mediating ECM disturbances and the progression of myxomatous mitral valve disease using the syndromic *Fbn1^C1039G/+^* mouse model and in vitro systems of primary porcine VICs. We show that SMA, an actin binding protein, is important for ECM changes consistent with myxomatous degeneration, and its expression is regulated by mechanical tension. These novel insights could inform the development of future non-surgical therapeutics to halt the progression of mitral valve degeneration thereby avoiding end-stage prolapse.

## 2. Materials and Methods

### 2.1. Mice

*Fbn1^C1039G/+^* (B6.129-Fbn1tm1Hcd/J) mice and *Fbn1^+/+^* wild type littermates were purchased from Jackson Labs (Stock No: 012885) and maintained as previously described [20]. *Fbn1^C1039G/+^* males were bred with *Fbn1^+/+^* wild type females resulting in the F1 generation of experimental *Fbn1^C1039G/+^* mice and *Fbn1^+/+^* wild types that were used as controls. Animals were genotyped to determine the presence of the mutant (~212 bp) or wild type allele (~164 bp) by PCR amplification of genomic DNA and complementary primers (Fbn1FOR: CTCATCATTTTTGGCCAGTTG; Fbn1REV: GCACTTGATGCACATTCACA). Thirty cycles of PCR were performed (95 °C for 30 s, 59 °C for 45 s, 72 °C for 40 s), and products were separated on a 2% agarose gel. All animal procedures were approved by institutional guidelines established by the Medical College of Wisconsin under the approved IACUC protocol AUA0006769.

### 2.2. Histological Analysis of Murine Tissue

Whole hearts from postnatal day 1 (PND1), 10 week and 12-month *Fbn1^C1039G/+^* or wild type (*Fbn1^+/+^*) mice were fixed overnight in 4% paraformaldehyde (PFA), diluted in 1X Phosphate Buffered Saline (PBS) at 4 °C and processed for paraffin embedding. Seven-μm-thick sections were cut and mounted on Fisherbrand Colorfrost Plus glass microscope slides (Thermo Fisher Scientific, Inc., Waltham, MA, USA), deparaffinized in a graded ethanol series and subject to histological analysis. For immunofluorescence, tissues were subjected to antigen retrieval by boiling for 10 min (mins) in Antigen Unmasking Solution, Citric Acid Based (Vector Laboratories, Burlingame, CA, USA) followed by 30 min additional incubation at room temperature. Following incubation, tissue sections were blocked in 1% bovine serum albumin, 0.1% cold water fish skin gelatin, 0.1% Tween 20, and 0.05% NaN_3_/PBS for 1 h at room temperature. For immunohistochemistry, to detect the percentage of SMA-positive cells, tissues were single-stained with anti-SMA (A2547 Monoclonal Anti-Actin α-Smooth Muscle clone 1A4, ascites fluid, 1:400, Sigma-Aldrich Co., St. Louis, MO, USA). For subsequent experiments, to detect SMA and extracellular matrix components, double staining was performed with anti-SMA (A2547 Monoclonal Anti-Actin α-Smooth Muscle clone 1A4, ascites fluid, 1:400, Sigma-Aldrich Co.), and Collagen Ia1 (Col1a1) (ab34710, Rb pAb to Col1a1 1:100, Abcam, Cambridge, United Kingdom), or anti-SMA, (ab5694, Rb pAb to SMA, 1:200, Abcam) and Chondroitin Sulfate Proteoglycan (CSPG) (C8035, Monoclonal Anti-Chondroitin Sulfate antibody produced in mouse, clone CS-56, ascites fluid, 1:200, Sigma-Aldrich Co.). Double staining was also performed using anti-SMA and MLK1/MRTF-A (14760, Rabbit Ab, 1:200, Cell Signaling Technology, Danvers, MA, USA). Primary antibodies were incubated together with tissue sections overnight at 4 °C. The following day, slides were washed in 1X PBS to remove non-specific binding and subsequently incubated for 1 h at room temperature in Alexa Fluor 488 or 568 goat anti-mouse IgG (Invitrogen by Thermo Fisher Scientific, Inc., Carlsbad, CA, USA). Slides were washed with 1X PBS and mounted with Vectashield HardSet Antifade Mounting Medium with DAPI (Vector Laboratories, Inc.). Adjacent tissue sections were subjected to Russell-Movat Pentachrome staining (American MasterTech Scientific, Inc., Lodi, CA, USA) according to the manufacturer’s instructions. Images of PND1, 10 week and 12 month old hearts were visualized using an Olympus BX51 microscope and captured using Cell Sens software, or the EVOS M7000 (Invitrogen by Thermo Fisher Scientific, Inc.).

### 2.3. Quantitation of Histological Analysis

**Figure 1:**
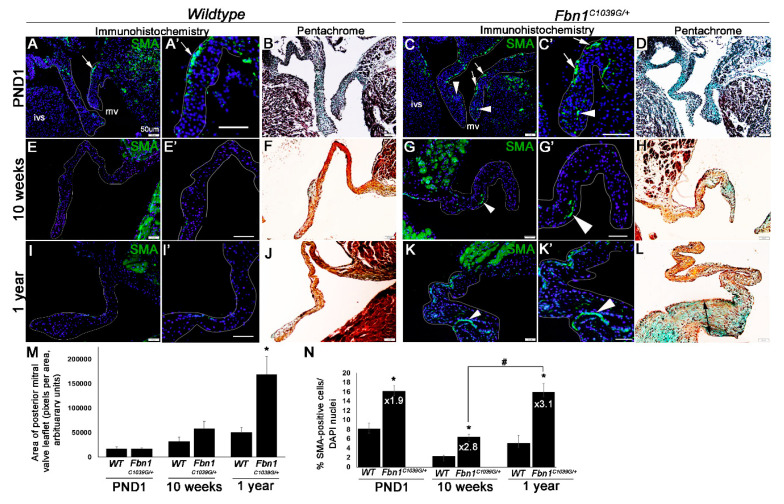
α-smooth muscle actin (SMA) expression increases over time in myxomatous valves from *Fbn1^C1039G/+^* mice. (**A**,**C**,**E**,**G**,**I**,**K**) Representative images of SMA (green) immunofluorescent staining in the interstitium of mitral valves from wild type (WT) (**A**,**E**,**I**) or *Fbn1^C1039G/+^* pups (**C**,**G**,**K**) at postnatal day 1 (PND1) (**A**–**D**); 10 weeks (**E**–**H**) and 1 year (**I**–**L**) of age, respectively. Higher magnification insets are shown in (**A’**,**C’**,**E’**,**G’**,**I’**,**K’**). White arrows (**A**,**C**) indicate a developmental population of SMA-positive valve interstitial cells (VICs) in the proximal hinge region of WT (**A**) and *Fbn1^C1039G/+^* mice (**C**) at PND1. White arrowheads (**C**,**G**,**K**) indicate ectopic SMA immunoreactivity in *Fbn1^C1039G/+^* mice; (**B**,**D**,**F**,**H**,**J**,**L**) Pentachrome staining of mitral valve tissue sections of WT (**B**,**F**,**J**) and *Fbn1^C1039G/+^* mice (**D**,**H**,**L**); (**M**) Average area of posterior mitral valve leaflet taken from 2D tissue sections at PND1, 10 weeks, and 1 year from WT and *Fbn1^C1039G/+^* mice, *n* = 3, * *p* > 0.05, using one-way ANOVA comparing *Fbn1^C1039G/+^* mice to WT controls at each time point; (**N**) percentage and fold change of SMA-positive cells (over DAPI-positive nuclei) in *Fbn1^C1039G/+^* mice compared to WT, at each time point, *n* = 3, * *p* > 0.05, using one-way ANOVA comparing *Fbn1^C1039G/+^* mice to WT controls at each time point. # for Figure 1N indicates statistical significance, *p* > 0.05 as indicated.

As shown in Figure 1, the area of the posterior mitral valve leaflet was calculated for *n* = 3 *Fbn1^C1039G/+^* or wild type mice at PND1, 10 weeks or 1 year of age using ImageJ 1.52q software (NIH). The average percentage of SMA-positive cells was calculated by counting the number of SMA-immunoreactive cells, divided by the number of cell nuclei as determined by DAPI staining for 3 sections on the same slide, then averaging these values to obtain the average number of SMA-positive cells in all sections on that slide. This was repeated for 2 additional slides, and then the average number of SMA-positive cells for each of the 3 slides was used to calculate the average percentage of SMA-positive cells, such that 9 total tissue sections of mitral valve leaflets from 3 biological replicates of *Fbn1^C1039G/+^* or wild type mice were analyzed. Findings were reported as a fold change in SMA-positive cells in the *Fbn1^C1039G/+^* compared to wild type controls at each time point. Data for the mitral valve leaflet area and average percentage of SMA-positive cells were analyzed using ordinary one-way ANOVA with Tukey’s multiple comparisons test for multiple groups to compare the mean of each genotype at each timepoint to the mean of every other timepoint. For the quantitation of SMA-positive cells in the aortic valve at 10 weeks of age, 3 sections from 3 different slides per mouse were quantified, with 3 mice utilized per genotype, and a total of 9 sections each of *Fbn1^C1039G/+^* or wild type mice were analyzed. For the aortic valve at PND1, one section each from 3 mice of each genotype (*Fbn1^C1039G/+^* or wild type) were analyzed. Findings were reported as percentage of Green Fluorescent Protein (GFP) (SMA)-positive cells, and subjected to an unpaired, two-tailed Student’s t-test comparing percentages of SMA-positive cells in the *Fbn1^C1039G/+^* and wild type mice at the 10 week and PND1 timepoints.

**Figure 2:**
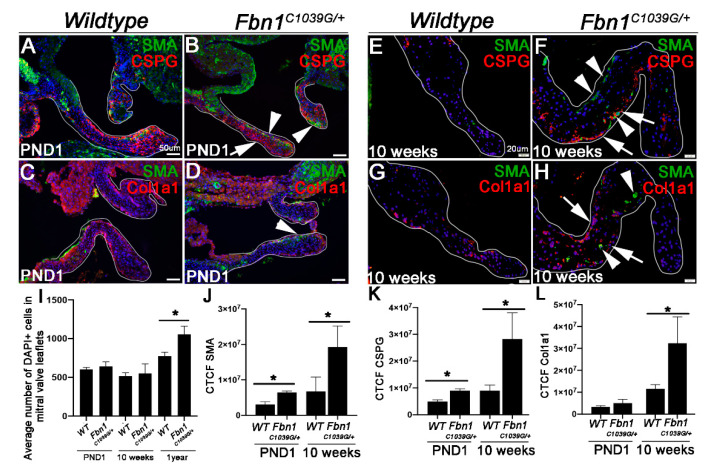
Extracellular matrix markers of myxomatous degeneration are increased in *Fbn1^C1039G/+^* mice from postnatal day 1 (PND1) and associated with ectopic α-smooth muscle actin (SMA) expression. SMA (green) and chondroitin sulfate proteoglycan (CSPG, red) immunofluorescent co-staining in mitral valves (outlined in white) from wild type (WT) (**A**,**E**) and *Fbn1^C1039G/+^* mice (**B**,**F**) at postnatal day 1 (PND1) (**A**,**B**); and 10 weeks (**E**,**F**) of age. (**C**–**H**) Double staining of SMA (green) with Col1a1 (red) in mitral valves from wild type (WT) (**C**,**G**) and *Fbn1^C1039G/+^* mice (**D**,**H**) at postnatal day 1 (PND1) (**C**,**D**); and 10 weeks (**G**,**H**) of age. White arrowheads indicate SMA immunoreactivity, arrows highlight CSPG or Col1a1 staining; (**I**) average number of DAPI-positive nuclei in mitral valve leaflets from *Fbn1^C1039G/+^* mice and WT littermates at each time point indicated, *n* = 3, * *p* > 0.05, using one-way ANOVA in *Fbn1^C1039G/+^* mice compared to WT controls; (**J**–**L**) Corrected Total Cell Fluorescence (CTCF) quantification of SMA (**J**); CSPG (**K**) and Col1a1 (**L**) immunoreactivity in mitral valve leaflets from *Fbn1^C1039G/+^* mice and WT controls at PND1 and 10 weeks. *n* = 3, * *p* > 0.05, using a Student’s t-test in *Fbn1^C1039G/+^* mice compared to WT controls.

In Figure 2, the average cell number of each complete mitral valve section was calculated as the average total number of cells in *n* = 3 complete mitral valves of *Fbn1^C1039G/+^* or wild type mice at each time point, as indicated by DAPI nuclear staining. Corrected total cell fluorescence (CTCF) to measure immunoreactivity was calculated using ImageJ 1.52q software (NIH). CTCF was calculated as the Integrated Density—(area of mitral valve leaflet visible in each image X Mean background readings) for each individual image. All CTCF values for each part of the mitral valve were added together to compute the total CTCF for the entire mitral valve replicate. Finally, CTCF was reported as the average CTCF of 3 complete mitral valve sections in *n* = 3 *Fbn1^C1039G/+^* or wild type mice. Data for the average cell number were analyzed using ordinary one-way ANOVA with Tukey’s multiple comparisons test. An unpaired, two-tailed Student’s t-test was used to analyze the average CTCF at either the PND1 or 10 week time point comparing the *Fbn1^C1039G/+^* to the wild type mice. All data are plotted as the mean with standard deviation.

### 2.4. Cell Culture

Porcine mitral valve interstitial cells (pmVICs) were isolated as previously described [30], released at passage 2 and cultured to confluency in Dulbecco’s Modification of Eagle’s Medium (DMEM, Corning) with 10% Fetal Bovine Serum (FBS, Atlanta biologicals) and 1% Pen Strep (Gibco). Cells from passage 4 to passage 6 were used for all subsequent experiments. Following treatments, pmVICs were harvested for protein extraction or fixed for immunofluorescence (see below).

For SMA siRNA experiments, pmVICs were seeded at 250,000 cells per well in BioLite 6-well plates in complete medium. The next day, cells were serum-starved for 16 h, then either transfected with 75 nM ON-TARGET plus SMART pool siRNA to porcine SMA (Dharmacon GE Healthcare Life Sciences) or transfected with a siGLO RISC-Free Control (Horizon Discovery Ltd., Waterbeach, United Kingdom) using Lipofectamine 3000 Reagent (Thermo Fisher Scientific, Inc.) in 2% FBS-supplemented media. The cells were further incubated for 96 h.

For Latrunculin A treatment, pmVICs were seeded at 250,000 cells per well in BioLite 6-well plates in a complete medium. The following day, cells were treated with either 100 ng/mL of Latrunculin A (Molecular Probes, Inc., Eugene, OR, USA) or Dimethylsulfoxide (DMSO, ATCC) for 36 h.

To target MRTF inhibition, pmVICs were seeded at 250,000 cells per well in BioLite 6-well plates in a complete medium for 24 h. Cells were then treated with 50 μM of CCG-203971 (Sigma-Aldrich Co.) or DMSO for 24 h in 2% FBS media.

For comparative stiffness studies, pmVICs were seeded at 250,000 cells per well in 6-well Softwell hydrogel plates coated with Collagen I at 2 and 25 kPa substrate stiffness (Matrigen) or BioLite tissue culture plastic plates (Thermo Fisher Scientific, Inc.) for 24 h.

### 2.5. Cell Immunofluorescence

Following treatments, pmVICs were washed with 1× PBS then fixed for 30 min in 0.1% glutaraldehyde at room temperature. Cells plated on different substrate stiffnesses (2 and 25 kPa) were fixed in 4% PFA for 30 min at room temperature. After 2 additional 1× PBS washes, cells were subjected to immunofluorescent block as described above. For stiffness experiments, cells were then incubated with anti-SMA overnight at 4 °C. Treated cells were subjected to incubation with two primary antibodies against SMA and Decorin (ab137508 Rb pAb to Decorin, 1:200, Abcam) overnight at 4 °C. The following day, cells were washed in 1× PBS and incubated in Alexa Fluor 488 goat anti-mouse IgG to detect SMA or Alexa Fluor 568 donkey anti-rabbit IgG to detect Decorin for 1 h at room temperature. After additional PBS washes, cells were mounted in respective wells with Vectashield HardSet Antifade Mounting Medium with DAPI. Imaging was performed using the EVOS M7000 with three images taken per well in *n* = 3 wells.

The average pmVIC number following treatments was determined as the average number of DAPI-positive nuclei in *n* = 3 images in 3 independent cultures. CTCF for each immunoreaction was calculated for all pmVICs in the field of view of the image as described above and presented as the average CTCF normalized to cell number of 3 images for *n* = 3 wells. Data for the average cell number, and CTCF values for SMA and Decorin were analyzed using an unpaired, two-tailed Student’s t-test to compare the treated group to the control group within each different treatment. All data are plotted as the mean with standard deviation.

### 2.6. Western Blot

At the conclusion of experiments, treated pmVICs and cells grown on 25 kPa and tissue culture plastic substrates were washed with 1X PBS, then lysed on ice in 1X Cell Lysis Buffer (Cell Signaling Technology) supplemented with Pierce EDTA-Free Protease Inhibitor Mini Tablets (Thermo Fisher Scientific, Inc.), and protein concentrations were determined via the Pierce BCA Protein Assay Kit (Thermo Fisher Scientific, Inc.). Cell lysates at concentrations from 5 to 22 μg underwent SDS-polyacrylamide gel electrophoresis and were transferred onto Nitrocellulose membranes (Invitrogen by Thermo Fisher Scientific, Inc.) via the iBlot Dry Blotting System (Life Technologies). To detect SMA protein levels, 5–10 μg of protein was used, and to detect all other protein levels, between 10 and 22 μg of protein was used. Membranes were then washed with 1X TBS with Tween (TBST, Tris Base, NaCl, pH 7.6) and blocked in 5% milk (blotting-grade blocker, nonfat dry milk, Bio-Rad) in 1X TBST for 1 h at room temperature. Following block, membranes were incubated with antibodies against SMA (1:20,000), Col1a1 (1:1000), or MRTF-A (1:1000) in 5% Bovine Serum Albumin (BSA, Fisher BioReagents) in 1X TBST, overnight at 4 °C. The following day, membranes were washed with 1X TBST, incubated with either Anti-mouse IgG Horse Radish Peroxidase (HRP)-linked antibody (Cell Signaling Technology) or Anti-rabbit IgG HRP-linked antibody (Cell Signaling Technology) in 5% milk for 1 h at room temperature. Following washing in 1X TBST, proteins were detected via chemiluminescence as indicated by SuperSignal West Pico Chemiluminescent Substrate, Pierce ECL Western Blotting Substrate or SuperSignal West Femto Maximum Sensitivity Substrate (Thermo Fisher Scientific, Inc.) and visualized via the iBright FL1000 imaging system (Thermo Fisher Scientific, Inc.). Membranes were subsequently stripped and incubated with anti-GAPDH (14C10, 1:2000, Cell Signaling Technology) to assess loading. Bands indicating SMA (~42 kDa) and Col1a1 (doublet at ~130 and 150 kDa) were quantified via ImageJ software and normalized to the calculated GAPDH levels (~37 kDa) for *n* = 9 replicates in the SMA siRNA treated cells and *n* = 3 replicates in the Latrunculin A treated cells. For the CCG-203971 experiments, SMA, Collagen I, and MKL1/MRTF-A (~145 kDa) bands for *n* = 3 replicates were quantified via ImageJ software and normalized to GAPDH. Finally, for mechanical stiffness experiments, SMA and MKL1/MRTF-A bands were quantified and normalized to GAPDH for *n* = 3 replicates. For SMA siRNA experiments, data were analyzed as the percentage change in protein expression of SMA and Col1a1 by calculating the level of SMA or Col1a1 in treated cells as a percentage of the average level of SMA or Col1a1 in the control cells set at 100%. For the Latrunculin A, CCG-203971, and stiffness experiments, data were analyzed as the average protein intensity normalized to the GAPDH of the treated group compared to the control group, or 25 kPa compared to tissue culture plastic (TCP) in the stiffness experiments, for SMA, Col1a1, or MRTF-A. All data underwent an unpaired, two-tailed Student’s t-test to compare the treated group to the control group, or 25 kPa to the TCP group, and were plotted as the mean with standard deviation.

## 3. Results

### 3.1. α-Smooth Muscle Actin (SMA) Expression Increases in Affected Mitral Valves of Fbn1^C1039G/+^ Mice during the Progression of Myxomatous Degeneration

Human and canine myxomatous valves isolated at the end-stage of disease are known to have increased SMA expression compared to healthy controls [31,32,33]. However, the temporal onset of this phenotype relative to myxomatous changes in the ECM has not been studied. To examine this, we utilized the *Fbn1^C1039G/+^* mouse model that has previously been shown to exhibit thickened mitral valves by PND6.5 and develop systolic prolapse by 9 months of age [20]. From RNA-seq data, we have shown that Fbn1 is highly expressed in heart valves throughout life [34] and based on previous studies, it is anticipated that mRNA and protein levels do not change in mitral valves from *Fbn1^C1039G/+^* mice, but rather microfiber formation is impaired [22]. As shown in Figure 1A–D, mitral valve leaflets from *Fbn1^C1039G/+^* pups at PND1 were grossly indistinguishable from wild type (*Fbn1^+/+^*) littermate controls (Figure 1B,M) by Pentachrome staining. However, immunohistochemistry detected an increase in the number of cells expressing SMA within the interstitial compartment of the mitral valve leaflets of *Fbn1^C1039G/+^* pups (Figure 1A,C,N). Consistent with our previous work [4], there was a small number of SMA-expressing cells along the atrial surface of the mitral valve leaflet in wild type pups that were likely remnant of embryonic development (arrow, Figure 1A). However, there were additional cells expressing SMA throughout the leaflets of *Fbn1^C1039G/+^* pups (arrowheads, Figure 1C). By 10 weeks, there was a subtle but insignificant increase in mitral valve leaflet thickness in *Fbn1^C1039G/+^* mice compared to the controls (Figure 1G,H compared to Figure 1E,F,M). However, the fold change in SMA-immunoreactive cells was significantly increased at this time point (Figure 1E,G,N). Worthy of mention, the percentage of SMA-positive cells in *Fbn1^C1039G/+^* mice at 10 weeks was less than at PND1. This was attributed to the remaining development cells after birth (Figure 1N) which disappeared by young adult stages (Figure 1E). However, the fold change of SMA expression was higher in *Fbn1^C1039G/+^* mice compared to the wild type controls at 10 weeks (×2.8) compared to at PND1 (×1.9) (Figure 1N). By 1 year, mitral valves from *Fbn1^C1039G/+^* mice were notably thick and dysmorphic with myxomatous changes in the ECM as determined by Pentachrome staining (Figure 1K,L,M), with a further increase in SMA expression and fold change (Figure 1K,N). Incidentally, SMA-positive cells were also increased in aortic valves from *Fbn1^C1039G/+^* mice at PND1 (×2.1) and 10 weeks (×7.5). Not surprisingly, the percent of SMA-positive cells was higher in affected *Fbn1^C1039G/+^* aortic valves at both time points, compared to mitral valves likely due to known aortic defects (data not shown). Together, these data showed that increases in SMA expression were observed by PND1 in mitral valves from *Fbn1^C1039G/+^* pups prior to significant myxomatous changes. Furthermore, the increase in SMA expression over wild type controls continued to increase throughout the duration of disease pathogenesis.

### 3.2. Ectopic SMA Expression Is Associated with Myxomatous ECM Changes within the Belly Region of the Mitral Valve Leaflets

Previous studies have shown that mitral valves from *Fbn1^C1039G/+^* pups at PND6.5 are thickened and exhibit systolic prolapse by 9 months [20]. However, the development of pathogenesis during this time has not been reported. Marfan Syndrome-associated MVP is accompanied by myxomatous changes in the ECM as indicated by an abnormal accumulation of proteoglycans, largely decorin, versican and lumican [9,10,17,35] and changes in collagen fibers including increased expression [15]. To examine myxomatous degeneration relative to ectopic SMA expression, immunohistochemistry was performed on *Fbn1^C1039G/+^* pups at PND1 and adult mice at 10 weeks, representative of early and mid-stages of disease, respectively. Consistent with Figure 1, SMA immunoreactivity was increased in mitral valves from *Fbn1^C1039G/+^* pups compared to the wild type controls at PND1 (Figure 2A–D,J) without any significant changes in overall cell number (Figure 2I). In addition, increased SMA was associated with higher chondroitin sulfate proteoglycan (CSPG) expression (Figure 2A,B,K), but no significant changes in collagen 1a1 (Col1a1) (Figure 2C,D,L). By 10 weeks, SMA-positive cells were notably localized within the mid “belly region” of the mitral valve leaflet from *Fbn1^C1039G/+^* mice (arrowheads, Figure 2F,H,J) and associated with increased accumulation of CSPG, and perhaps more extensively Col1a1 (Figure 2F,H,K,L).

### 3.3. Reduced SMA Expression by siRNA, or Actin Depolymerization in Porcine Mitral Valve Interstitial Cells Leads to Decreased Expression of Col1a1 and Decorin

Ectopic SMA expression in VICs has been previously described in myxomatous mitral valve disease; however, its role in mediating ECM changes is not known. To examine this, we utilized a porcine mitral VIC (pmVIC) culture system published by our group [30] and targeted SMA knockdown by siRNA. As a readout of ECM changes relative to myxomatous degeneration described in human disease, increased expression of Collagen1a1 (Col1a1) and the proteoglycan Decorin were examined.

**Figure 3:**
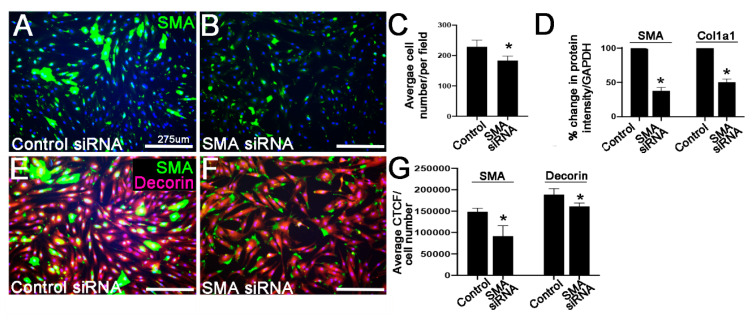
Reduction in α-smooth muscle actin (SMA) by siRNA in porcine mitral valve interstitial cells (pmVICs) results in decreased Collagen I (Col1a1) and Decorin expression. (**A**,**B**) Immunohistochemistry of SMA in pmVICs treated with 75 nM control or SMA-targeted siRNA for 96 h; (**C**) average number of DAPI-positive nuclei in treated pmVICs, *n* = 3, * *p* > 0.05 using a Student’s t-test in SMA-siRNA treated cells compared to control-siRNA; (**D**) percent change in protein intensity following Western Blot analysis of SMA (5 μg protein blot) and Col1a1 (20 μg protein blot) expression normalized to GAPDH in pmVICs treated with SMA siRNA compared to controls, *n* = 9, * *p* > 0.05 using a Student’s t-test; (**E**,**F**) immunohistochemistry of SMA (green) and Decorin (red) in pmVICs treated with 75 nM control or SMA-targeted siRNA for 96 h; (**G**) Corrected Total Cell Fluorescence (CTCF) quantification of SMA and Decorin immunoreactivity normalized to cell number, *n* = 3, * *p* > 0.05 using a Student’s t-test.

Culturing VICs on a stiff substrate increases SMA expression [36,37]. Therefore, the following experiments were performed by culturing pmVICs on tissue culture plastic and treating with 75 nM ON-TARGET plus SMART pool SMA-siRNA and control siRNA for 96 h. SMA siRNA disrupted overall VIC morphology, and treated cells were smaller, less elongated, and lacked their typical spindle-shape (Figure 3A,B,G). In addition, SMA siRNA led to a small decrease in pmVIC number, suggesting decreased viability (Figure 3C). Using the siRNA approach, SMA was reduced by ~62.24 ± 4.87% as determined by Western Blot, and this was associated with a ~49.53 ± 4.63% decrease in Col1a1 (Figure 3D). Immunoreactivity analysis of the proteoglycan Decorin was similarly decreased ~14.65 ± 4.30% in SMA siRNA-treated pmVICs (Figure 3E,F,G).

**Figure 4:**
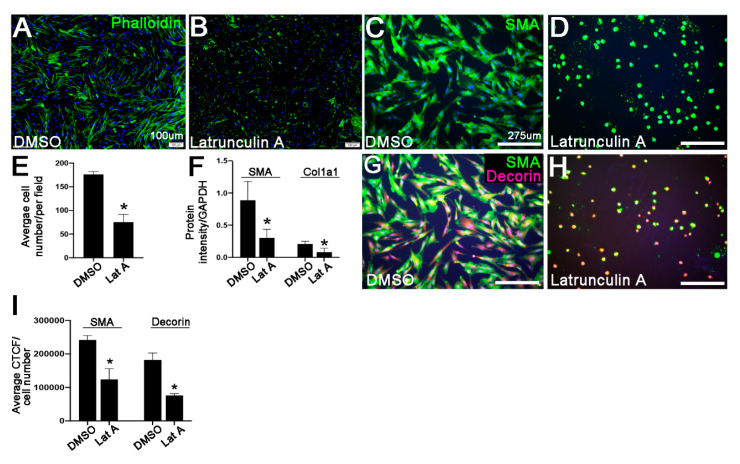
Reduction in functional α-smooth muscle actin (SMA) by Latrunculin A treatment in porcine mitral valve interstitial cells (pmVICs) results in decreased Collagen I (Col1a1) and Decorin expression. (**A**,**B**) Phalloidin staining to highlight F-actin organization in pmVICs treated with DMSO vehicle control (**A**) or 100 ng/mL of Latrunculin A (**B**) for 36 h; (**C**,**D**) SMA immunoreactivity in pmVICs treated with DMSO (**C**) or Latrunculin A (**D**); (**E**) average number of DAPI-positive nuclei in pmVICs treated with DMSO or Latrunculin A (Lat A), *n* = 3, * *p* > 0.05 using a Student’s t-test in Latrunculin A treated cells compared to DMSO controls; (**F**) quantitation of protein intensity following Western Blot analysis of SMA (10 μg protein blot) and Col1a1 (10 μg protein blot) expression normalized to GAPDH in pmVICs treated with Latrunculin A (Lat A) compared to DMSO, *n* = 3, * *p* > 0.05 using a Student’s t-test; (**G**–**H**) immunohistochemistry of SMA (green) and Decorin (red) in pmVICs treated with DMSO (**G**) or Latrunculin A (**H**); (**I**) Corrected Total Cell Fluorescence (CTCF) quantification of SMA and Decorin immunoreactivity normalized to cell number, *n* = 3, * *p* > 0.05 using a Student’s t-test.

In many cell types including myofibroblasts, SMA is assembled into actin filaments [38]. Therefore, to determine if disrupting actin filaments could influence SMA and subsequent Col1a1 and Decorin expression, pmVICs were treated with 100 ng/mL Latrunculin A, or DMSO vehicle for 36 h. As expected, Latrunculin A as an actin depolymerizing compound, significantly disrupted the cytoskeleton of pmVICs (Figure 4A,B), and resulted in mislocalization and decreased expression of SMA (Figure 4C,D,F). In addition, cell viability or density was reduced (Figure 4E). As a result of this targeting approach, Western Blot analysis showed that Col1a1 was reduced (Figure 4F), in addition to Decorin (Figure 4G–I) as determined by quantitative analysis of immunoreactivity. Together, these data suggest that SMA is in part, required in VICs for the remodeling of the ECM consistent with myxomatous degeneration, including secretion of collagens and proteoglycans.

### 3.4. Rho/MRTF/SRF Signaling Mediates ECM Remodeling in Cultured Mitral Valve Interstitial Cells

**Figure 5:**
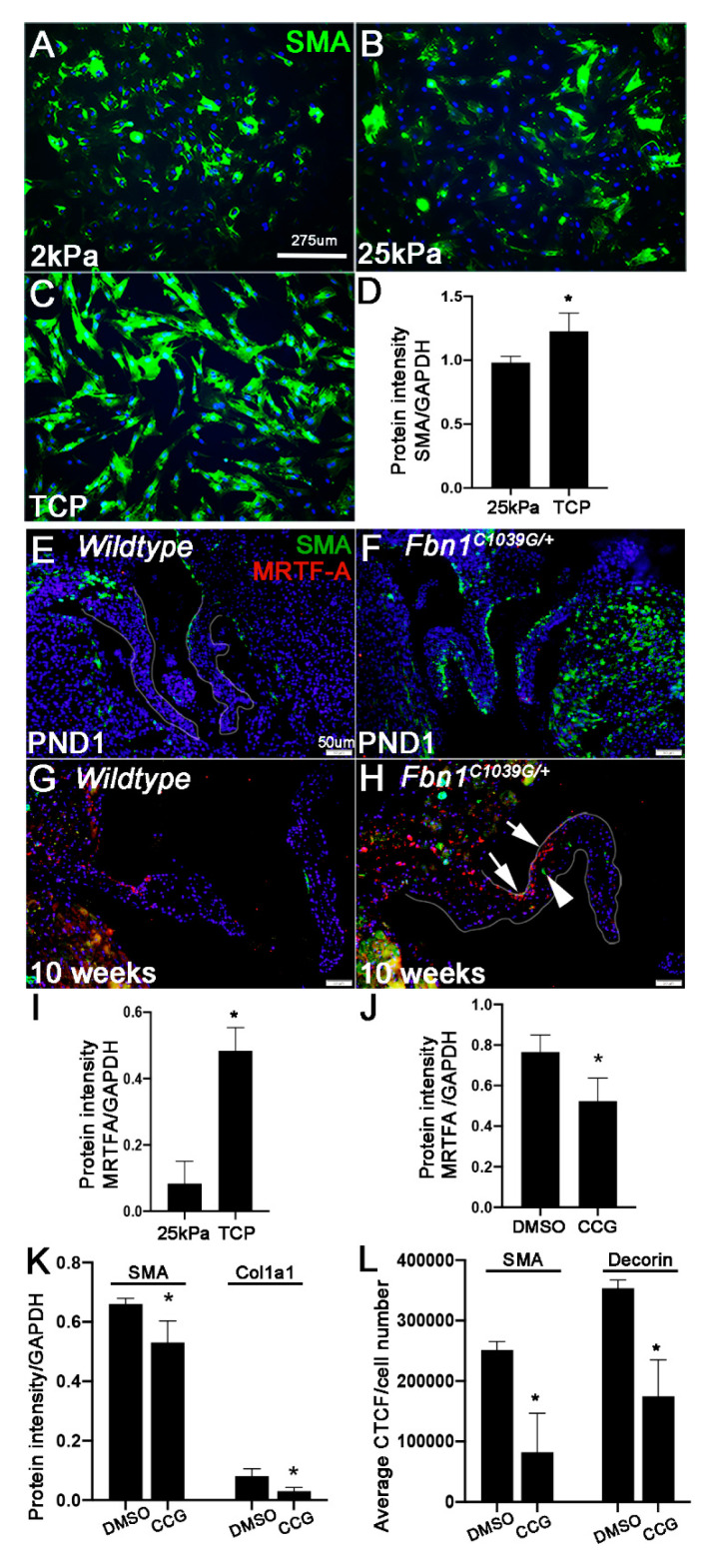
In valve interstitial cells, increased SMA expression is in part, mediated by Rho/MRTF/SRF mechano-sensitive signaling to regulate extracellular matrix (ECM) homeostasis. (**A**–**C**) Immunohistochemistry of SMA in porcine mitral valve interstitial cells (pmVICs) cultured on 2 kPa (**A**), 25 kPa (**B**), and tissue culture plastic (TCP) (**C**) for 24 h. (**D**) Quantitation of protein intensity following Western Blot analysis of SMA (5 μg protein blot) in lysates collected from pmVICs cultured on 25 kPa and tissue culture plastic (TCP) for 24 h normalized to GAPDH, *n* = 3, *p* > 0.05 using a Student’s t-test. (**E**–**H**) immunohistochemistry of SMA (green) and MRTF-A (red) in tissue sections of mitral valves from wild type (WT) (**E**,**G**) or *Fbn1^C1039G/+^* (**F**,**H**) mice at postnatal day 1 (PND1) (**E**,**F**) and 10 weeks (**G**,**H**) of age. (**I**) quantitation of densitometry analysis of Western Blot findings to determine changes in MRTF-A expression (22 μg protein blot) normalized to GAPDH in pmVICs cultured on 25 kPa and TCP, *n* = 3, * *p* > 0.05 using a Student’s t-test. Rho/MRTF/SRF signaling was inhibited by treating pmVICs with 50 μM of CCG-203971 or DMSO vehicle control for 24 h. (**J**,**K**) Quantitation of protein intensity following Western Blot analysis of MRTF-A (**J**) (18 μg protein blot), SMA (5 μg protein blot), and Collagen I (Col1a1) (**K**) (18 μg protein blot) in pmVICs treated with CCG-203971 compared to DMSO controls, *n* = 3, * *p* > 0.05 using a Student’s t-test. (**L**) Corrected Total Cell Fluorescence (CTCF) quantification of SMA and Decorin immunoreactivity in CCG-203971 treated pmVICs compared to DMSO controls and normalized to cell number, *n* = 3, * *p* > 0.05 using a Student’s t-test.

SMA as an actin isoform, is modulated by mechanical tension in many cell types including cardiac fibroblasts [39]. Consistent with others [36,37], we showed that SMA expression and spreading of VICs increased when cultured on higher substrate stiffnesses (2 and 25 kPa, Tissue Culture Plastic) for 24 h (Figure 5A–D). In cardiac fibroblasts, mechanical tension regulates SMA through MRTF signaling, whereby it translocates to the nucleus to bind SRF-dependent transcription factors including SMA (reviewed [40]). While it is known that prolapsed mitral valves have a global reduction in tissue stiffness [18,19], myxomatous degeneration is not uniform, and based on our studies in mice (Figure 2), ectopic SMA and changes in ECM appear to be regionalized around the mid belly region. Therefore, to determine if SMA-positive cells in this location are associated with changes in mechanical tension, MRTF-A expression was examined by immunohistochemistry in *Fbn1^C1039G/+^* pups at PND1 and mice at 10 weeks. As shown, MRTF-A expression was not robustly expressed at PND1 in mitral valves from either *Fbn1^C1039G/+^* pups or the wild type controls (Figure 5E,F). However, by 10 weeks, there was an association between increased MRTF-A expression (arrows, Figure 5H) and SMA (arrowhead, Figure 5H) in the belly region of affected mitral valves. MRTFA was also significantly increased in pmVICs cultured on TCP compared to a softer 25 kPa substrate (Figure 5I). To determine the requirement of Rho/MRTF/SRF signaling for SMA expression and ECM remodeling in cultured pmVICs, cells were treated with 50 μM CCG-203971 for 24 h which reduced MRTFA expression by ~31.54 ± 14.79% (Figure 5J). Following this, we observed a small, but significant decrease in SMA, as well as Col1a1 (Figure 5K) and Decorin (Figure 5L), suggesting that mechanosensitive signaling might play a role in mediating SMA expression and ECM changes in VICs.

## 4. Discussion

MVP as a result of myxomatous degeneration is commonly presented in human patients of connective tissue disorders, and for severe cases, surgical valve repair or replacement is required. Previous studies reporting increased SMA in a subset of VICs in human and canine myxomatous valves utilize tissue removed during surgical repair or replacement, and therefore, the phenotype is often overtly severe and end-stage, which may not reflect the true pathogenesis [31,32,33]. As a result, the contribution of “activated” SMA-positive cells to myxomatous pathogenesis over time has not been examined, and furthermore, the mechanisms underlying increased SMA in affected valves have remained unclear. Using a murine model of human Marfan Syndrome (*Fbn1^C1039G/+^*) and complementary in vitro assays, we provide initial insights into the involvement of SMA-positive VICs to the progressive deterioration of the mitral valve ECM structure during early stages of disease through end-stage functional decline.

Idiopathic myxomatous degeneration and MVP are seen as progressive deterioration manifested during the later stages of life. Syndromic, and potentially non-syndromic mitral valve disease, have a genetic component and phenotypes develop early during embryogenesis or early stages of childhood [7,41]. Here, we show that affected mitral valves from syndromic *Fbn1^C1039G/+^* mice begin to thicken around 10 weeks of age (Figure 1D,H,M), although the original paper reported thickening from as early as PND6.5 [20]. During these early postnatal stages of mitral valve disease, gross structural defects were not observed, and ECM organization appears largely unaffected, although we observe increased (chondroitin sulfate) proteoglycan (Figure 2K). These subtle changes are accompanied by significant ectopic SMA expression in VICs (Figure 1C,N and Figure 2J), suggesting that this phenotype is not merely an effect of end-stage disease, but its expression is initiated during the early stages of pathology prior to excessive myxomatous degeneration. At this time, the phenotype of SMA-positive cells in myxomatous valves has not been well defined and further work is needed. However, based on studies in other systems, it is suggested that these cells represent a subset of ”activated” VICs that exhibit a myofibroblast-like profile and subsequently remodel the ECM. In addition, as described in calcified aortic valves, SMA could also be marking a smooth muscle cell fate [42], and this is supported in part by observations showing co-localization of SMA with SM22 in a subset of cells from *Fbn1^C1039G/+^* mitral valves (data not shown).

Studies of canine myxomatous mitral valve disease have suggested that SMA expression is the product of endothelial-to-mesenchymal transformation (EMT) [43]. However, in mouse models of myxomatous degeneration, cell tracing experiments have not detected active EMT [44]. Therefore, we anticipate that SMA is induced by alternative mechanisms in murine mitral valve disease. The Fbn1 microfibril niche provides an important environment for growth factor signaling, and loss of function prevents Tgfβ sequestration. As a result, human patients harboring the *C1039T* (C1039G equivalent) mutation exhibit elevated levels of circulating Tgfβ [20,23,45]. Consistently, in *Fbn1^C1039G/+^* mice, Tgfβ signaling (as indicated by pSmad2) is increased in mitral valve tissue at PND6.5. Furthermore, antagonism of the Tgfβ pathway by treatment with a neutralizing antibody during embryonic development (E14.5–E17.5) prevents the valve pathology observed at postnatal stages [20]. In hand with this observation, independent studies have shown that Tgfβ positively regulates SMA expression in VICs [46]. Based on these data, we believe that early ectopic SMA expression in VICs and the initiation of myxomatous degeneration begin during embryonic development and is driven by increased Tgfβ activity as a result of the underlying genetic mutation in *Fbn1^C1039G/+^* pups. It is also noted that SMA expression is observed along the atrial surface of the developing mitral valve in wild type pups (Figure 1A), and this is considered to be remnant from embryonic development and required for physiological remodeling of the ECM during postnatal maturation [4].

As discussed, SMA is observed in VICs at end-stage MVP, and here we show in *Fbn1^C1039G/+^* mice that SMA expression temporally increases throughout disease progression. Therefore, in addition to elevated Tgfβ, there are likely additional factors driving the continued elevation of SMA. Previous studies and Figure 5A–D show that in VICs, SMA is regulated by mechanical tension [36,37]. While myxomatous degeneration in the valve ECM, including increased proteoglycans and collagen fiber rearrangement that together lead to a global reduction in tissue stiffness [18,19], degeneration at least in *Fbn1^C1039G/+^* mice, is not uniform (Figure 2E–H). In Figure 2E–H, we note increased CSPG and Col1a1 immunoreactivity localized within the mid belly region of the mitral valve leaflet of *Fbn1^C1039G/+^* mice at 10 weeks, and we speculate that these ECM changes could result in regionalized differences in mechanical stiffness. In addition, abnormal increased SMA expression in VICs in this active mid belly region is associated with increased expression of the mechanoregulator, MRTFA (Figure 5H). However, it is also noteworthy that there are several SMA-positive cells that do not express MRTF-A, and likewise it appears that in other cells, MRTF-A is not well associated with SMA expression, suggesting in part, independent roles. However, experiments in Figure 5 highlight the requirement of Rho/MRTF/SRF signaling for SMA expression in VICs (Figure 5K), and antagonism also attenuates collagen and proteoglycan expression (Figure 5K,L). Based on these data, it is suggested that over time, myxomatous degeneration leads to regionalized differences in the mechanical stiffness of the valve ECM. In turn, areas of higher tension (belly region), potentially as a result of increased Col1a1, activate mechano-sensitive pathways (MRTF) to further induce SMA expression and exacerbate myxomatous degeneration to the point of mechanical failure or prolapse, as described, by 9 months of age [20]. However, additional work is required to confirm this.

To date, the observation of increased SMA expression in myxomatous valves at end-stage disease is correlative, and it is not clear if SMA-positive cells are the cause, or effect of pathological ECM disruption, or even the direct result of the *Fbn1* mutation. In this study, SMA was targeted directly by siRNA (Figure 3), or indirectly by actin depolymerization (Figure 4), and findings commonly show that Decorin and Col1a1 are attenuated when SMA expression is reduced. These findings provide insights into a contributing role of SMA-positive VICs to the onset and progression of myxomatous degeneration. However, additional studies are required to fully characterize the phenotype and function of these abnormal cells during the progression of degenerative mitral valve disease.

## 5. Conclusions

In summary, this study focuses on better understanding a highly prevalent disorder that affects over 176 million people worldwide. At present, end-stage surgical repair or replacement is the only effective treatment for MVP sufferers, and this comes with insuperable complications and no guarantee of long-term success, especially in the growing pediatric population [5,8,47]. Findings from this study have shed light on the temporal regulation of SMA in a sub-population of VICs during the progression of myxomatous mitral valve degeneration, and our work supports a causative role for these abnormal cells in promoting disease phenotypes. From these studies, we can begin to postulate how these findings can be applied to the development of new, mechanistic-based therapies to target the abnormal activation of VICs with the goal of halting disease progression, thereby avoiding the insuperable complications of end-stage surgical intervention.
